# Canine Coronavirus Highly Pathogenic for Dogs

**DOI:** 10.3201/eid1203.050839

**Published:** 2006-03

**Authors:** Canio Buonavoglia, Nicola Decaro, Vito Martella, Gabriella Elia, Marco Campolo, Costantina Desario, Massimo Castagnaro, Maria Tempesta

**Affiliations:** * University of Bari, Bari, Italy;; † University of Padua, Padova, Italy

**Keywords:** Coronavirus, dog, mortality, dispatch

## Abstract

Canine coronavirus (CCoV) is usually responsible for mild, self-limiting infections restricted to the enteric tract. We report an outbreak of fatal disease in puppies caused by a pathogenic variant of CCoV that was isolated from organs with severe lesions.

Coronaviruses are large, enveloped, positive-stranded RNA viruses (*1*). Three different coronaviruses have been identified in dogs (*2**,**3*). Canine coronavirus (CCoV) type I and type II are included in group 1 coronaviruses, and their evolution is related to that of feline coronavirus (FCoV) type I and type II. FCoV type II originated by heterologous recombination between CCoV type II and FCoV type I, while CCoV type I is genetically more similar to FCoV type I than to CCoV type II (*3*). In addition, 2 FCoV biotypes that differ in pathogenicity have been observed in cats.

The onset of acute fatal disease (feline infectious peritonitis) is caused by pantropic variants (able to disseminate throughout the organism) of enteric FCoVs with deletions or recombinations in the 3c and 7b genes at the 3´ end of the FCoV genome (*4*). Similarly, changes in tissue tropisms in porcine and murine coronaviruses (*5**,**6*) and adaptation of the recently recognized severe acute respiratory syndrome–associated coronavirus (*7*) to humans have been related to mutations or deletions. A third canine coronavirus, CRCoV, detected in the respiratory tract, has <96.0% amino acid (aa) conservation in the spike (S) protein with bovine coronavirus within group 2 coronaviruses, which provides strong evidence for a recent host-species shift (*2*).

Coronavirus infection in dogs is usually restricted to the enteric tract. The infection is self-limiting and in general produces only mild or asymptomatic forms of enteritis (*8*). We report the identification of a pantropic, highly pathogenic variant of CCoV type II.

## The Study

In May 2005, a severe outbreak of fatal systemic disease occurred in a pet shop in Bari, Italy. Clinical symptoms were initially observed in 3 miniature pinschers (45 days of age) and 1 cocker spaniel (53 days of age) and consisted of fever (39.5°C–40°C), lethargy, inappetence, vomiting, hemorrhagic diarrhea, and neurologic signs (ataxia, seizures) with death after 2 days. The same symptoms were observed 3–4 days later in 2 other miniature pinschers (45 days of age) and 1 Pekinese (56 days of age). Necropsy of the dogs showed hemorrhagic enteritis, abundant serosanguineous fluid in the abdominal cavity, and severe lesions in the parenchymatous organs. The lungs had multiple, patchy, red areas of consolidation. Livers were yellow-brown and congested, with hemorrhages on their surfaces, and spleens were enlarged with subcapsular hemorrhages. Variable gross changes in other organs included multifocal hemorrhagic renal cortical infarcts and petechial hemorrhages on lymph node surfaces.

**Figure 1:**
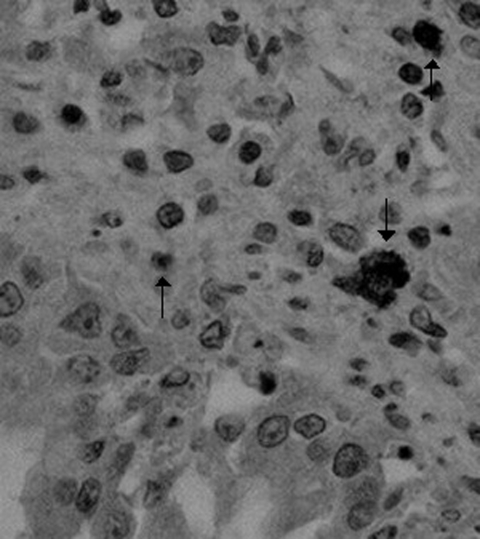
Immunohistochemical detection of canine coronavirus antigen (arrows) in canine lung tissue by a specific monoclonal antibody (magnification ×400).

Virologic and bacteriologic investigations on the parenchymatous organs did not detect common canine pathogens, notably canine parvovirus type 2, canine distemper virus, canine adenovirus type 1 and type 2. CCoV type I and type II were identified in the intestinal contents of all puppies by genotype-specific real-time reverse transcription–polymerase chain reaction (RT-PCR) assays (*9*). CCoV type II RNA was also detected in lungs (median 1.08 × 10^6^ RNA copies/μL of template), spleen (median 4.46 × 10^6^ RNA copies/μL of template), liver (median 9.02 × 10^4^ RNA copies/μL of template), kidney (median 7.54 × 10^4^ RNA copies/μL of template), and brain (median 5.23 × 10^3^ RNA copies/μL of template). Virus-induced cytopathic effect was observed in A-72 cells, and CCoV type II strain (CB/05) was isolated from all tissues examined except brain tissue. Immunohistochemical analysis with a CCoV-specific monoclonal antibody detected CCoV antigen in the organs with gross lesions that were examined (lungs, kidneys, liver, spleen, gut, and lymph nodes) (Figure 1).

**Figure 2:**
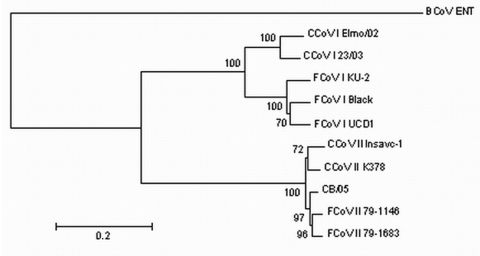
Neighbor-joining tree of the spike protein of canine coronavirus (CCoV) and feline coronavirus (FCoV). The following reference strains were used for phylogenetic analysis: CCoV type I strains Elmo/02 (GenBank accession no. AY307020) and 23/03 (AY307021); CCoV type II strains Insavc-1 (D13096) and K378 (X77047); FCoV type I strains KU-2 (D32044), Black (AB088223) and UCD-1 (AB088222); FCoV type II strains 79-1146 (X06170) and 79-1683 (X80799); and bovine coronavirus (BCoV) strain ENT (NC_003045). The tree is rooted on BCoV-ENT and drawn to scale. A statistical support was provided by bootstrapping >100 replicates. The scale bar represents 20 substitutions per 100 sequence positions.feline coronavirus (FCoV). The following reference strains were used for phylogenetic analysis: CCoV type I strains Elmo/02 (GenBank accession no. AY307020) and 23/03 (AY307021); CCoV type II strains Insavc-1 (D13096) and K378 (X77047); FCoV type I strains KU-2 (D32044), Black (AB088223) and UCD-1 (AB088222); FCoV type II strains 79-1146 (X06170) and 79-1683 (X80799); and bovine coronavirus (BCoV) strain ENT (NC_003045). The tree is rooted on BCoV-ENT and drawn to scale. A statistical support was provided by bootstrapping >100 replicates. The scale bar represents 20 substitutions per 100 sequence positions.

The sequence of the 3´ end of the genome (8.8 kb) of the pantropic CCoV strain was determined by RT-PCR amplification and sequencing of overlapping fragments. The S, envelope, and membrane proteins and nucleoprotein showed a high degree of amino acid identity with the cognate open reading frame (ORF) of CCoV type II. The S protein of strain CB/05 had the highest identity to FCoV type II strain 79-1683 (Figure 2). Comparison with strain CB/05 was possible only with CCoV type II strains Insavc-1 (*10*) and BGF (*11*) and CCoV type I strains Elmo/02 and 23/03 (*3**,**12*) because of a lack of data on the 3´ end of the CCoV genome in the genes encoding for nonstructural proteins (NSPs) 3a, 3b, 3c, 7a, and 7b. NSPs 3a, 7a, and 7b were not altered. NSP 3b (22 aa) was 49 aa shorter than expected because of a 38-nucleotide deletion and a frame shift mutation in the downstream sequence that introduced an early stop codon. NSP 3c (244 aa) was 6 aa shorter and 79 aa longer than the cognate proteins of the enteropathogen strain BGF and the attenuated strain Insavc-1a, respectively.

To confirm the pathogenic potential of strain CB/05, we experimentally infected two 6-month-old dogs (authorization no. 67/2002-C released by Ministry of Health of Italy). Two milliliters of cryolysate of a lung-derived first-passage virus in A-72 cells were administered intranasally to the dogs. The cell cryolysate tested negative for other common canine pathogens and had a 50% tissue culture infectious dose of 10^5.50^/50 μL on A-72 cells and 1.18 × 10^7^ RNA copies/μL of template by real-time RT-PCR. The virus was reisolated from the experimentally infected dogs. Severe clinical symptoms characterized by pyrexia (temperature 39.8°C–40.1°C), anorexia, depression, vomiting, diarrhea, and leukopenia were observed that persisted 8–10 days. Despite the severe symptoms, the dogs slowly recovered from their illness.

## Conclusions

Point mutations or deletions in the S protein and NSPs have been associated with changes in tropism and virulence of coronaviruses (*4**–**7**,**13*). CCoV strain CB/05 showed intact structural and nonstructural proteins, with an S protein closely related to that of other type II CCoVs. The only striking change was the truncated form of NSP 3b. Whether the deletion in the ORF of NSP 3b is involved in pathobiologic changes should be assessed with reverse genetic systems.

The present study describes for the first time the occurrence of fatal infections in dogs by coronaviruses. Experimental infection of dogs with the virus isolate resulted in a severe systemic disease that mimicked the clinical symptoms observed in the outbreak. However, the different ages at infection (6 months vs. <2 months) likely resulted in the disease being nonfatal. Accordingly, the appearance of pathogenic CCoV variants should always be regarded as a potential threat to domestic dogs and considered when unexplainable fatal disease outbreaks occur in puppies.

Epidemiologic studies are required to determine whether the pantropic CCoV strain is a new coronavirus variant emerging in canine populations or a widespread infectious agent of dogs that usually goes undetected. Vaccination trials could also help determine whether the CCoV vaccines currently available are effective against the highly virulent CCoV strain.

The 2002–2003 SARS epidemic has demonstrated that the study of animal coronaviruses is paramount to understanding the ecology and evolution of human coronaviruses. The coronaviruses of carnivores provide a paradigmatic model of how coronaviruses cross the species barriers, adapt to new host species, and change their pathogenicity.
